# British Dental Association

**Published:** 1885-11

**Authors:** 


### The British Dental Association.

The annual general meeting of the British Dental Association was held at Cambridge on the 27th of August. A large number of members attended and altogether the meeting was most successful.

From the secretary's report we perceive that a new branch of the Association called the Central Counties has been formed.

Three successful prosecutions have been carried on under the Dentists' Act and other persons were induced to discontinue their infringement of the Act without legal proceedings.

A member of the association was expelled for unprofessional conduct, and the General Medical Council has on the representations of the Executive removed the name of a person convicted of fraud from the Dentist's Register.

The Board recommended that the next president elect should be Sir Edwin Saunders.

It was suggested that in order to secure a better attendance of members the meeting should be held a week earlier than heretofore.

The President in his valedictory address dealt mainly with the legal position of the profession in England, in the following words:

Taking a comprehensive view of the mass of offenders against the Dentists' Act, they seem to be divisible into two separate and materially different classes; first, those who are registered, and, it may be, specially qualified practitioners, but who conduct themselves in a manner unworthy of their position; and second, those who are not registered, but who engage in practice as if they were duly registered practitioners. And it is in dealing with the latter class—those whose offense consists merely in carrying on practice without any right to that registration which alone entitles them to do so—that, perhaps, the greatest difficulty is experienced. In these cases no range or grade of penalties exists. In the other class, that of registered practitioners guilty of professional indecorums, the punishment, I presume, as in the case of medical practitioners, shades off from suspension or withdrawal of a diploma or erasure from the Register, down to a mere remonstrance or admonition. In the other it is the summary and total ablation and extinction of the culprit, which is, in all cases, sought, and is the award. This constitutes a difficulty, which, though serious, has occasionally almost a touch of humor in it, since, even after such supposed extermination, the offender may, like the phœnix of old, be found rising from his ashes—possibly in a new, but most likely in a form and shape as defiant and offensive as ever. Prosecution after prosecution may here be advocated and persisted in, but in such miserable cases as these generally are, there exists the danger of this procedure being construed into one of persecution, the risk of such misconstruction being increased by the fact that the part of public prosecutor has to be undertaken by a private individual and a brother in trade. In such cases the ignorance of the real question at issue, and the interest, notwithstanding, taken in them by that great judicial body and court of law—the general public—plays even a more important and decisive part, and one of greater weight in certain ways, than the statements or opinions of the members of that profession more immediately concerned. For we must recollect that in such cases the public is ever inclined to "gently scan our brother man," and, moreover, is ever inclined also to stamp it as beneath the dignity of the learned professions to descend to fight what it is pleased to term, those petty indiscretions, which are alleged to brand the perpetrator in the public eye much deeper, much sooner, and much more effectually, when he is let alone. And there is no doubt that such a feeling being known to be abroad, is, to a greater or less extent, a consideration which acts as an embarrassment and a restraint in a certain measure upon the normal course of justice.

I am not here, however, upholding a policy of non-interference with offenders—I am not dissuading from legal measures or proceedings being instituted against law-breakers. Far from it; I merely question whether the game is worth powder and shot; I am attempting to inculcate that perhaps in certain instances some of these cases, although irritating, are of so insignificant and contemptible a character as to render it questionable whether it might not be more expedient, more dignified, and more efficacious that they should be set at nought. In dealing with the other class of offenses, especially with those of a more flagrant character, little hesitation need be felt. Foremost among these, the assumption of false or spurious titles, of pretended qualifications, of misleading and fictitious subterfuges of any kind, constitute the individual simply an impostor who under covert and convenient seeming, is as dangerous to the general community, as his conduct is nefarious towards the duly constituted practitioners alongside of him. Of infamous conduct in a professional point of view, this is, perhaps, the worst and most momentous form, and one to which no consideration or lenience need be shown. Apart from such more flagrant cases, however, there are gradations of disreputable or unprofessional conduct more difficult to dispose of. There are questions of unprofessional conduct between the shades of which no exact or definite line has yet been drawn. To do so is very difficult. Take the case of advertisements. Now you are aware that the committal of any offense which amounts to a misdemeanour, or any conduct of an infamous nature in a professional point of view—certainly a somewhat vague definition—may, according to the 13th section of the Dentists' Act be followed by erasure from the Register; that same erasure from the Register again entailing, peremptorily and *ipso facto*, the withdrawal of the diploma by the authority from whom it may have been granted many years before, and perhaps obtained, not only creditably, but with honors and distinction.

This enactment, however, seems to be about all that has been provided for in such cases, and the severe and serious nature of such a sentence renders its infliction painful and repulsive, and, therefore, except in extreme circumstances, explains its always being difficult to obtain, either at the hands of the Medical Council or of the Licensing Boards. Such a system of punishment, in fact, has of late been brought under notice as a supremely interesting subject of study, and one not in every case at all likely to be unanimously approved of. Punishments of this kind, indeed, where a second penalty is entailed solely in virtue of, and altogether consequent upon the mere fact of a previous one having been inflicted, are in the sense of a recent article by Hon. Justice Stephen, appearing, I think, in the *Nineteenth Century Review*, always undesirable and hampering to the judge, and generally distasteful, as operating with retrospective severity, and tending to punish literally for having deserved well in earlier life.

Let me, however, not wander into abstract or tedious speculations. If I have expatiated upon a subject likely to be a moot and debatable point, it is because it forms the key-note and leading matter of my text, as found in the resumé given of the Association's work last year, while it is at the same time a subject demanding dispassionate consideration, and a full insight into the difficulties with which it is beset. And I merely go thus far towards keeping in mind that whether or not such difficulties are unremovable or insurmountable, still they exist, and are to be taken into account. Great differences of opinion on such questions necessarily exist in all such bodies as the British Dental Association; various estimates are certain to be held of the magnitude and importance of different forms of offending and various ideas entertained as to the severity and summary and off-hand nature of the punishment which ought to be inflicted. It is as well, however, that our action in such cases should be as little as possible of a vindictive, or high-handed, or headlong tendency, but rather with the disinterested aim and object, as Sir John Lubbock well put it in one of the earlier debates upon the Dentists' Bill of protecting the public against quacks and quackery, by giving it a complete and easily accessible opportunity of ascertaining for itself whether or not anyone assuming to be a trustworthy member of the dental profession is properly qualified; and this, there is no doubt, is already largely supplied in the publication of the Dental Register. And the duty of the dental profession, more especially as it is represented in the British Dental Association, is to assist in maintaining that Register in as pure and reliable a state as possible.

A vote of thanks was proposed by Mr. J. Tomes, F. R. S. and seconded by Sir E. Saunders.

The President acknowledged the vote, after which he vacated the chair in favor of his successor Mr. Richard White, who delivered the inaugural address, which, omitting some paragraphs of only local interest we append.

Although at present there are but few of our members who can rejoice in calling this University their Alma Mater, rest assured the day is not far distant when many dentist's in *statu pupillari* will enter the great medical school established here.

The general and professional education of the dental practitioner ought not to be inferior to that of his at present more favoured medical confrere. The medical council being of this opinion with regard to general education requires the same preliminary examination marks in both cases.

Our social progress will be commensurate with our educational advancement, of which indeed it must be the natural outcome for mental culture brings social qualifications of the highest order, and these qualifications are duly recognized by the existing code of civilization.

In the future men who are ambitious of taking the best social position the dental profession affords will enter one of the Universities as students of medicine are doing at the present day. For a university career to a professional man offers immense advantages in after life. A status is gained, friendships are formed, and a healthy and beneficial tone is given to the character by the associations of the place (provided that the connections formed be happy and judicious), which, as years roll on, nothing appears to obliterate.

The social problem merges into the political, and the political prospect is favorable in direct proportion to the successful management and enlargement of the association whose primary aim is the faithful execution of the various provisions of the Dental Act, 1878.

We must regret the legislature did not appoint a public prosecutor to carry the cases of illegal practice into court, instead of requiring a private practitioner or an officer of the Association to become the prosecutor, thus placing the practitioner in a very invidious and painful position.

The members of the Association are under great obligations to those who boldly, and at the risk of much professional annoyance, have undertaken such a disagreeable duty.

We now come to the scientific forecast. The advancement of dentistry will depend largely on scientific investigation, by which I mean investigation executed on the basis of certain defined laws. The prospect is chiefly that of dental pathology and prophylactic dentistry or dental hygiene.

The histological anatomy of the teeth and their environment has been so thoroughly sifted in the recent past by the aid of the microscope that nature can have but few secrets to reveal in this direction. The physiology of the dental tissues has been established on a more or less satisfactory basis, leaving but a limited field of enquiry for future observers, but dental pathology is as yet in its infancy, and offers a magnificent prospect for original research. Many and varied are the theories regarding the etiology of caries, greater or less weight being given to the so-called external causes—mechanical, chemical and parasitic, including the action of the food, the buccal secretions, and air, while hardly sufficient attention has been bestowed on those more subtle internal agencies, developmental, nervous, vascular, and trophic. Moreover, we have heretofore intermingled predisposing and exciting causes. These must be isolated and duly classified before a satisfactory scientific basis can be established. How far parasitic germs can be the *materia morbi*, it is not for me to determine, but analogy suggests the probability of their being concomitant with the disease rather than the true exciting cause of decay. The air is ever impregnated with bacterial germs, and their nourishment being derived from the products of decomposition, the seats of decay offer a suitable nidus for their proliferation. In the impaired nervous force, the altered qualitative and quantitative blood supply, and trophic diminutions, we have what seem to be the truly predisposing causes of caries. The exact point of origin of the disease may be purely accidental, arising from the agency of any one or more of the so-called external causes, the importance of which must be, therefore, largely discounted. The elucidation of this complicated subject might be accelerated by experimental research, by ascertainimg the results of traction, section, and electrical irritation of the inferior dental nerve in animals, experimental alteration of the quantity of blood supplied to the teeth, and, where possible, the quality of the same.

The deterioration of the teeth, resulting from a departure from the laws of nature, has probably existed among civilized nations for thousands of years—for it is said that the inhabitants of the ancient empires of the world paid the same penalty from a like cause, if not to the same extent, that we are now doing. That caries exists in almost every mouth at the present day, and that its victims during the last few years have increased to an alarming extent, we do not doubt, for of that we have daily proof.

A question naturally arises—What physiological cause has brought about this change of structure? For it appears to be the only portion of the human frame that has exhibited this marked deterioration.

Those who have had the opportunity of examining many cases of the teeth of persons from four generations of patients (which has fallen to my lot) will I believe, acknowledge that the teeth in each succeeding generation, as a rule, have become more and more degenerated as regards their structure, and are more prone to be affected by caries, and that the teeth of females have much more deteriorated than those of the male sex; and, further, that through the female line this deterioration is much more marked than through the male line, for a mother with very defective teeth will invariably have children whose teeth partake of the same character. Acknowledging this deterioration may not an attempt be made to discover its cause?

That the mind of the mother exercises an immense influence over the constitution of the child when in its fœtal state no one can doubt. And it is not possible that in these days of constant exitement, and of general unrest among all classes, that its baneful effects may have an influence upon the rudimentary dental organs of the child at this early stage. And may not also in these days, the high mental pressure permitted, and the artificial life of early childhood, continuing in rebellion against nature's laws, carry on the degenerative process that had been so early commenced?

The result of an immoral life, handed down from generation to generation, tells with fearful effect upon the children of such parents. And the medical treatment in former times to combat the resultant disease—if not at the present day—has had its influence upon the formation of the teeth.

The diet of children during their early years, and until the teeth are fairly formed, has been considered of great importance, although in some cases where this matter has been most strictly adhered to, the result has not always, I fear, proved satisfactory. It appears to me that much cannot be accomplished in this important subject until we are in possession of statistics for our guidance, for without such assistance we are but groping in the dark.

Would it not be possible to ascertain certain facts from our patients which might be tabulated, from which data might be obtained that might guide us, as well as our medical friends, in advising such measures as might, to a certain extent combat the result of agencies that are at work in producing these deteriorated teeth.

Collective investigation in the hands of the British Medical Association has already largely assisted in unravelling the history of several obscure diseases, and if persevered in will aid in furthering the rational treatments of disease as opposed to the empirical drugging of the past.

Collective investigation would eminently assist us in ascertaining the causes of this general deterioration in the structure of the dental tissues. We might enquire regarding and record—

1. The age of patient;
2. The general health;
3. The number and general nature of the teeth;
4. The number diseased;
5. General health of parents and nature of their teeth;
6. If consanguinity existed;
7. Habits and diet of mother at time of birth;
8. Habits and diet of child in infancy and during juvenescence.

We thus arrive at the subject of prophylactic or preventive dentistry, or as it may be termed dental hygiene, that highest scientific branch of our profession in the future, which can alone be established as the outcome of the elucidation of the causes of disease, and will tend to place us higher in the estimation of the general public than the perfecting of any other branch of our art, for they will recognize in the advocacy of its principles that splendid abnegation of self which can have but one aim, the maintenance of the health of the community. We shall then work on the same line as our medical brethern, who seek to prevent disease as much as to combat it when already existing.

Papers were read by W. Spence Bate, on "Excision versus Extraction," Mr. Storer Bennett, on "Further Experiences with Herbst's Method of Gold Filling," by Dr. C. M. Cunningham, on "Cast Metal as a Base," by Mr. Oakley Coles, on "The Hopes and Fears of Dentistry," by Mr. Charters White, on "Section Cutting of Dental Tissues", and by Mr. Hutchinson, on "The Necessity of Teeth after Fifty Years of Age."

Clinics and demonstrations were given in the University Museum (Biological Laboratory).

### Books Received for Review.

C. H. Land. Scientific Adaptation of Artificial Dentures.
J. F. Flagg. Questions and Answers on Dental Pathology.
G. C. Crowley. Dental Bibliography.
M. W. J. Mrs. Letters on Children's Teeth, 3rd ed.

THE habit of observation, when once acquired, becomes as second nature, and an insight into the mystery of nature's workings is obtained, (we scarce know when or how) which amply repays us for the care and time we may bestow in the acquiring.

BUT, while counselling you to record, do not suppose I would urge you to that vulgar habit of rushing into print, our literature is already flooded with too much that is written, not because the author has that to impart which is worth the writing, but merely to gratify the vanity of him who writes.

STORER BENNETT.

